# Elamipretide for Barth syndrome cardiomyopathy: gradual rebuilding of a failed power grid

**DOI:** 10.1007/s10741-021-10177-8

**Published:** 2021-10-08

**Authors:** Hani N. Sabbah

**Affiliations:** Department of Medicine, Division of Cardiovascular Medicine, Henry Ford Hospital, Henry Ford Health System, 2799 West Grand Boulevard, Detroit, MI 48202 USA

**Keywords:** Elamipretide, Barth syndrome, Cardiomyopathy, Mitochondria

## Abstract

Barth syndrome is a rare and potentially fatal X-linked disease characterized by cardiomyopathy, skeletal muscle weakness, growth delays, and cyclic neutropenia. Patients with Barth syndrome are prone to high risk of mortality in infancy and the development of cardiomyopathy with severe weakening of the immune system. Elamipretide is a water-soluble, aromatic-cationic, mitochondria-targeting tetrapeptide that readily penetrates and transiently localizes to the inner mitochondrial membrane. Therapy with elamipretide facilitates cell health by improving energy production and inhibiting excessive formation of reactive oxygen species, thus alleviating oxidative stress. Elamipretide crosses the outer membrane of the mitochondrion and becomes associated with cardiolipin, a constituent phospholipid of the inner membrane. Elamipretide improves mitochondrial bioenergetics and morphology rapidly in induced pluripotent stem cells from patients with Barth syndrome and other genetically related diseases characterized by pediatric cardiomyopathy. Data with elamipretide across multiple models of disease are especially promising, with results from several studies supporting the use of elamipretide as potential therapy for patients with Barth syndrome, particularly where there is a confirmed diagnosis of cardiomyopathy. This review highlights the challenges and opportunities presented in treating Barth syndrome cardiomyopathy patients with elamipretide and addresses evidence supporting the durability of effect of elamipretide as a therapeutic agent for Barth syndrome, especially its likely durable effects on progression of cardiomyopathy following the cessation of drug treatment and the capability of elamipretide to structurally reverse remodel the failing left ventricle at the global, cellular, and molecular level in a gradual manner through specific targeting of the mitochondrial inner membrane.

## Introduction

Barth syndrome is a rare and potentially fatal X-linked disease characterized by cardiomyopathy, skeletal muscle weakness, growth delay, and cyclic neutropenia [1]. It is caused by defects in TAZ, a gene whose product, tafazzin, is an enzyme essential for cardiolipin biosynthesis and remodeling—cardiolipin is an integral component of normal mitochondrial function [2–4]. Patients with Barth syndrome are at high risk of death during infancy related to the development of cardiomyopathy and severe weakening of the immune system [5]. The estimated incidence of Barth syndrome is 1:300,000–400,000 births with 111 patients in the USA and 230–250 globally, although the disease is accepted as being underdiagnosed [5]. There is an unmet need for the development of an effective therapy for patients with Barth syndrome.

Elamipretide is a water-soluble, aromatic-cationic, mitochondria-targeting tetrapeptide that readily penetrates and transiently localizes to the inner mitochondrial membrane [6]. Elamipretide therapy facilitates cell health by improving energy production and inhibiting oxidative stress caused by excessive reactive oxygen species (ROS) formation and, as such, is a promising agent for the treatment of patients with Barth syndrome. It targets mitochondrial dysfunction in energy-starved myocytes, crossing the outer mitochondrial membrane and associating with cardiolipin, a key phospholipid of the inner mitochondrial membrane [7]. Elamipretide improves left ventricular ejection fraction (LVEF), lowers left ventricular end-diastolic pressure, reduces cardiomyocyte and ventricular hypertrophy, limits myocardial fibrosis, and improves mitochondrial ATP-generating capacity in animal models [8–10]. These attributes of elamipretide help position the compound as a potential therapeutic agent targeting numerous metabolic cardiomyopathies, notably that associated with Barth syndrome.

This review highlights the challenges and opportunities presented in treating Barth syndrome and the potential role of elamipretide. It will address the experimental evidence supporting the durability of effect of elamipretide as a potential therapeutic agent for Barth syndrome, especially its likely durable effects on disease progression following the cessation of drug treatment. Finally, the review will also address the capability of elamipretide to structurally reverse remodel the failing left ventricle at the global, cellular, and molecular level in a gradual temporal manner through its specific and selective effect on the mitochondrial network.

## Barth syndrome cardiomyopathy

Cardiomyopathies comprise a group of diseases that affect the heart muscle. They are either confined to the heart or are part of a generalized systemic myopathy disorder, both of which often lead to cardiovascular death or progressive heart failure-related disabilities [11]. Cardiomyopathy is the single most frequent sign of Barth syndrome, occurring in approximately 90% of males with Barth syndrome, although its manifestation and severity varies among individuals [12, 13]. Several cardiomyopathic phenotypes have been described with Barth syndrome. Dilated cardiomyopathy is common and is characterized by decreased left ventricular systolic function, increased left ventricular mass, and increased left ventricular end-diastolic and end-systolic dimensions [12, 14, 15]. The dilated left ventricular phenotype in Barth syndrome may depend on the time course of the disease and may emerge as a late manifestation of disease progression. Data from the TAZPOWER trial [16] suggest that Barth syndrome is characterized by a small left ventricular volume consistent with restrictive cardiomyopathies and heart failure similar to that seen in young males with Duchenne muscular dystrophy before progression to dilation. A small left ventricular volume along with poor left ventricular filling leading to reduced stroke volume is also seen in patients with heart failure and preserved left ventricular ejection fraction (HFpEF). Consequently, any therapy that targets HFpEF may also elicit improvement in patients with Barth syndrome cardiomyopathy [16]. Left ventricular non-compaction is also commonly seen in Barth syndrome patients either alone or in conjunction with other cardiomyopathic phenotypes and is characterized by left ventricular trabeculations with associated left ventricular wall motion abnormalities [14]. Endocardial fibroelastosis may be seen, although less commonly [17]. Hypertrophic cardiomyopathy [18], as well as an apical form of hypertrophic cardiomyopathy [19], often characterized by small left ventricular volumes and poor left ventricular relaxation, is also reported to occur in Barth syndrome. A hypertrophic-dilated cardiac phenotype, characterized by thickening of the left ventricular walls combined with increased left ventricular mass and left ventricular end-diastolic dimension as well as depressed left ventricular systolic function, has also been reported [20]. Transition between distinct cardiomyopathy phenotypes has also been described in the setting of left ventricular non-compaction, which has been termed as an “undulating phenotype” [21]. No current mechanism has been proposed that explains the presence of the different cardiomyopathic phenotypes that have been observed in Barth syndrome. Evidence of different cardiomyopathy phenotypes is well documented in families with recognized sarcomeric mutations, suggesting a shared molecular etiology for the different forms of cardiomyopathy [22]. Additionally, there is an increased risk of cardiac arrhythmias in Barth syndrome, some of which may be life-threatening. The arrhythmias may be a direct result of abnormal mitochondrial function [23] and/or a function of the associated cardiac phenotype, as ventricular arrhythmias are well reported in dilated and hypertrophic cardiomyopathies [24].

Because mitochondrial dysfunction characterized by poor ATP production is integral to Barth syndrome and the cardiomyopathy phenotypes associated with Barth syndrome typically manifest poor left ventricular relaxation, the relationship between ATP and left ventricular relaxation is critical in understanding the disease process. For this reason, the importance of ATP for proper left ventricular relaxation and filling cannot be overemphasized. It is well documented that myocardial relaxation is an active, energy-dependent process [25]. Relaxation is typically described as having two phases: early active relaxation and late passive relaxation. The active relaxation during early diastole is energy (ATP) dependent; any depletion of ATP for whatever reason will therefore cause a slowdown in relaxation and hence poor left ventricular filling. Passive relaxation happens late in the diastolic phase during left atrial contraction and is dependent on the strength of atrial contraction but, more importantly, on compliance of left ventricular muscle. If left ventricular wall stiffness is increased, as in the presence of myocardial fibrosis, this phase of relaxation will not contribute to or will contribute very little to overall left ventricular filling. The obvious consequence of poor left ventricular filling is reduced forward stroke output or stroke volume, a hallmark of heart (pump) failure. It is important to note that many cardiomyopathies that occur in children and young adults can result in left ventricular dilation. In Barth syndrome, however, it appears that the left ventricle does not dilate much and LVEF is near normal, making most patients with this disease align within the defined boundaries of the HFpEF type of left ventricular failure rather than heart failure with reduced ejection fraction (HFrEF). In this scenario, increase of left ventricular end-diastolic volume (EDV), as was evident after treatment with elamipretide in Barth syndrome patients, should be viewed as a manifestation of improved left ventricular filling and a foundation for a higher stroke volume [25].

**Fig. 1:**
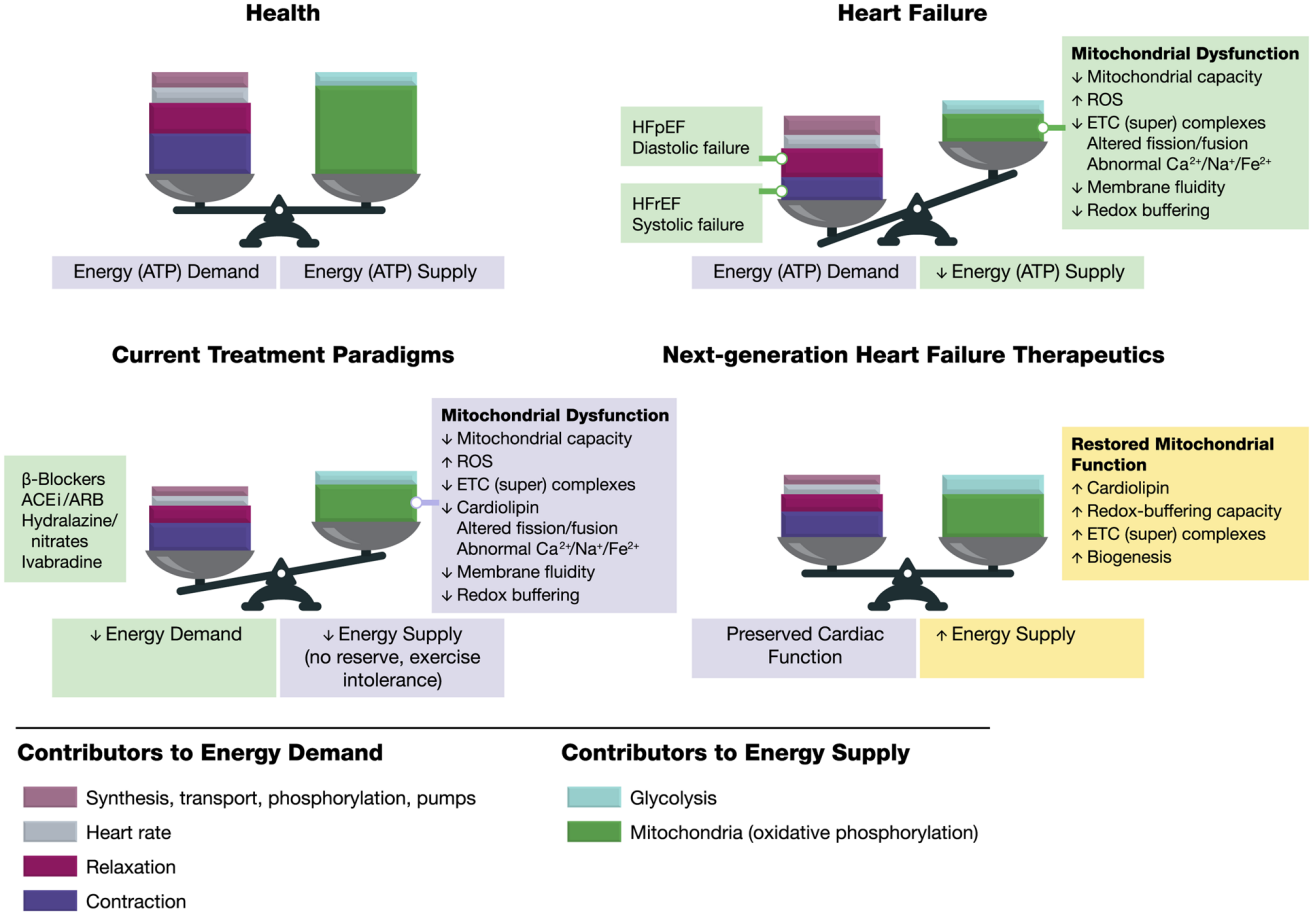
Factors affecting cardiac energy supply and demand [26]. In heart failure, the balance between cardiac demands and supply for energy is tipped such that the supply does not meet the demand. Inadequate myocardial energy production occurs during myocardial ischemia either at rest or following episodes of increased energy demand, causing left ventricular diastolic dysfunction. Reduced energy supply also accompanies HFrEF or HFpEF. Abnormalities in mitochondrial ultrastructure, dynamics, and enzymatic activities of key complexes of the electron transfer chain are common in the failing myocardium and are responsible, in large part, for the reduction in ATP production. Next-generation therapies can improve on existing standard-of-care therapies by bolstering mitochondrial energy production Adapted with permission from [26]. Abbreviations: *ACEi* angiotensin-converting enzyme inhibitor, *ARB* angiotensin II receptor blocker, ETC electron transport chain, *HFpEF* heart failure with preserved ejection fraction, *HFrEF* heart failure with reduced ejection fraction, *ROS* reactive oxygen species

Left ventricular diastolic dysfunction caused by inadequate myocardial energy production occurs during myocardial ischemia at rest or following episodes of increased energy demand states such as during exercise (Fig. 1). There is growing and compelling evidence that most forms of heart failure such as HFrEF or HFpEF are accompanied by a state of energy starvation (reduced energy supply). Abnormalities in mitochondrial ultrastructure, dynamics, and enzymatic activities of key complexes of the electron transfer chain (ETC) are common in the failing myocardium and are responsible, in large part, for the reduction in ATP production [9, 26–28]. A mismatch between capillary density and cardiomyocyte hypertrophy and impaired subendocardial perfusion due to increased left ventricular wall stress can also contribute to an imbalance between energy production and utilization in the failing heart. Because myocardial contraction and relaxation are both ATP-dependent processes, and because ATP availability is integral to cardiomyocyte calcium-reuptake, impaired left ventricular active relaxation and reduced left ventricular compliance are universal features of chronic heart failure [25].

## Mitochondrial dysfunction in heart failure

Mitochondria are double-membraned organelles that form the energy source or the “power grid” found in nearly all eukaryotic cells. Mitochondria function primarily to produce ATP from adenosine diphosphate (ADP), using macromolecular protein complexes that form the ETC [29]. Oxidative phosphorylation takes place along the inner mitochondrial membrane, during which reducing equivalents, e.g., nicotinamide-adenine dinucleotide (NADH) and reduced flavin adenine dinucleotide, are transferred from the carrier molecules to the ETC, while protons are pumped to the intermembrane space. Because the inner mitochondrial membrane is impermeable to most ions and small molecules, proton pumping generates a membrane potential that uses ATP synthase and inorganic phosphate to convert ADP to ATP [30, 31].

The protein complexes fixed along the mitochondrial inner membrane include NADH dehydrogenase (complex I), succinate dehydrogenase (complex II), cytochrome bc1 (complex III), and cytochrome c oxidase (complex IV) [29]. The heart possesses the highest content of mitochondria of any tissue in the body [26]. It comprises about 25% of cell volume in the human myocardium and approximately 35% of cardiomyocyte volume [29, 32, 33]. It has been shown that mitochondria-initiated programmed cell death or apoptosis is an important mechanism in heart failure [34]. Mitochondrial DNA and/or ROS are triggers of an inflammatory response in cardiac muscle [35–38]. Regulation of innate immunity by mitochondria has been increasingly recognized in both cardiac and non-cardiac diseases [35–39]. As sterile inflammation is common in heart failure, mitochondria have also emerged as an important pathogenic component. Collectively, these observations depict molecular mechanisms promoting the transition of mitochondria from having a purely energy-producing function to central hubs that influence cellular survival and death.

Cellular models for Barth syndrome including patient-derived fibroblasts, lymphoblasts, neutrophils, neonatal ventricular fibroblasts, and cardiomyocytes have been studied to reveal the morphological changes of mitochondria, the structural rebuilding of the respiratory chain, the decreasing respiratory capacity, and the increasing production of ROS [40–43]. In order to generate a human cellular model of Barth syndrome, somatic cells from Barth syndrome patients were used to generate induced pluripotent stem cells (iPSCs) with the capacity for self-renewal and differentiation. These cell models show a shift in the cardiolipin pool that recapitulates conditions observed in Barth syndrome patients. Further investigation of the iPSCs revealed that remodeling of the respiratory chain supercomplexes causes decreased mitochondrial respiration and increased ROS generation [44, 45]. These defects were also observed in cardiomyocytes generated by directed differentiation of pluripotent stem cells [45]. The data showed that induced stem cell-derived cardiomyocytes also recapitulated myopathic phenotypes.

## Current treatment for Barth syndrome cardiomyopathy

Treatment of cardiomyopathy in patients with Barth syndrome generally follows that for heart failure patients, including angiotensin-converting enzyme (ACE) inhibitors, angiotensin II receptor blockers, beta-blockers, and/or diuretics according to the particular heart failure phenotype [19]. Oral anticoagulation should be considered if appropriate [1]. Prostaglandin E_1_ may be beneficial with right ventricular failure [46]. Heart transplantation can be considered if heart failure treatment is ineffective [47–50] and bridging mechanical circulatory support can be used until transplantation becomes available [51].

## Elamipretide

A more logical approach to the treatment of Barth syndrome cardiomyopathy is to target the underlying mitochondrial pathology of Barth syndrome that precipitates the cardiomyopathy. Elamipretide is being investigated as such an approach.

Elamipretide is a water-soluble, aromatic-cationic, mitochondria-targeting tetrapeptide that readily penetrates and transiently localizes to the inner mitochondrial membrane where it associates with cardiolipin. Elamipretide improves inner membrane stability, protein–protein interactions, and ATP production, and reduces pathogenic ROS production. Cardiolipin plays an integral role in cristae formation, mitochondrial fusion, mitochondrial DNA stability and segregation, and organization of the respiratory complexes into supercomplexes for oxidative phosphorylation [52–56]. Elamipretide has been shown to enhance ATP synthesis in multiple organs and tissues, including the heart, kidney, neurons, and skeletal muscle [9, 52–55, 57]. High-resolution respirometry of ETC complexes in permeabilized ventricular fibers from ischemia–reperfusion rats showed that ischemia–reperfusion-induced decrements in mitochondrial complexes I, II, and IV were significantly alleviated with elamipretide [58]. Furthermore, studies in serial block-face scanning electron microscopy used to create high-resolution 3-dimensional reconstructions of cristae ultrastructure showed that disease-induced fragmentation of cristae networks was improved with elamipretide [58]. Studies using biomimetic membranes modeling of the inner mitochondrial membranes also showed that elamipretide improved mitochondrial inner membrane biophysical properties by aggregating cardiolipin [58]. Importantly, the elamipretide effect of improving membrane integrity was even observed when cardiolipin levels in the in vitro system were lowered to levels consistent with those seen in Barth syndrome [58]. Furthermore, the elamipretide-mediated effects on membranes were observed across independent model systems where cardiolipin was oxidized or enriched with monolysocardiolipin, the immature form of cardiolipin found in Barth syndrome [57, 59]. These studies suggest that mitochondrial structure and function are, as expected, interdependent and demonstrate that elamipretide targets mitochondrial membranes to stabilize cristae networks and improves bioenergetic function [58].

Elamipretide has been shown to improve mitochondrial bioenergetics and morphology rapidly (within hours or days) in iPSCs from Barth syndrome and other closely related pediatric cardiomyopathies [60, 61]. Re-establishing appropriate energy supply–demand balance (Fig. 1) leads to a restoration of healthy gene expression (within days) and improved cardiac and mitochondrial protein turnover (within weeks) [26, 62]. Many mitochondrial functions, including oxidative phosphorylation, are strongly associated with the inner mitochondrial membrane [59]. The abnormal structure and function of the mitochondrial network is reflected by impaired mitochondrial cristae structure and loss of mitochondrial network connectivity. Elamipretide has been shown by electron and fluorescence microscopy to restore this architecture in compromised mitochondria, resulting in improved cellular bioenergetics [58]. Studies using Barth syndrome patient-derived cell lines and iPSC-derived, beating cardiomyocytes from pediatric patients with cardiomyopathy showed that elamipretide also restores this architecture in compromised mitochondria, leading to improvements in overall cellular function [59]. Furthermore, in cells engineered using CRISPR to have a *TAZ* mutation, the decline seen in several different ETC subunits and altered mitochondrial quality control was attenuated after 7 days of elamipretide treatment in vitro [63].

### Preclinical studies of elamipretide on cardiac function in heart failure

A murine model of Barth syndrome has been developed [6]; however, the effects of elamipretide on cardiac function in heart failure in this model have not been reported. Elamipretide has been studied extensively in models of experimental left ventricular dysfunction and heart failure (Table 1). These studies in animal models of experimental heart failure provide much of the knowledge base available to date that supports the potential use of elamipretide for the treatment of patients with cardiomyopathies as in Barth syndrome.

**Table 1:** Summary of key preclinical studies of elamipretide in heart failure

Reference	Model	Results
Sabbah et al. [9]	Canine model of microembolization-induced advanced HF	Elamipretide (3 months) improved LVEF and indices of LV diastolic function, normalized plasma biomarkers (nt-proBNP, TNF-a, and CRP), SERCA2a activity, COX1 and ND1 DAMPs, and reversed mitochondrial abnormalities (respiration, ∆φm, maximum ATP synthesis rate, and ATP/ADP ratio) in LV myocardium compared to placebo
Eirin et al. [8]	Porcine model of renovascular hypertension manifesting HFpEF	Elamipretide (3 months) normalized mitochondrial respiration, mitochondrial calcium tolerance and permeability pore opening, mitochondrial membrane potential, SERCA2a activity, maximum of ATP synthesis rate, and mitochondrial complex I and IV activities, reduced ROS formation and cytochrome c release into the cytosolic compartment. Left ventricular relaxation was improved and cardiomyocyte hypertrophy reduced
Chiao et al. [64]	Old mice	Elamipretide (8 weeks) normalized diastolic functional deficit, increased Ea/Aa, and improved exercise tolerance with regression of cardiac hypertrophy accompanied by normalization of mitochondrial proton leak and ROS

*ADP* adenosine diphosphate, *CRP* C-reactive protein, *DAMP* DNA damage-associated molecular patterns, *Ea/Aa* early-to-late diastolic mitral annulus velocities, *HF* heart failure, *HFpEF* heart failure with preserved ejection fraction, *LV* left ventricle, *LVEF* left ventricular ejection fraction, *nt-pro-BNP* n-terminal pro-brain natriuretic peptide, *ROS* reactive oxygen species, *SERCA2a* sarco-/endoplasmic reticulum Ca^2+^ ATPase, *TNF-a* tumor necrosis factor-a, *∆φm* membrane potential

#### Elamipretide reverses SERCA2a maladaptations and ameliorates inflammatory markers in heart failure

Abnormal sarcoplasmic reticulum calcium cycling is a key characteristic of the failing heart along with a sustained increase of circulating levels of pro-inflammatory cytokines. These maladaptations can be viewed as resulting from biochemical alterations to cellular and molecular signaling pathways that result from the chronic heart failure state. Reversal of these abnormalities often requires long-term therapy capable of restoring aberranul cellular and molecular signaling. In a murine model of Barth syndrome, myocardial sarco-/endoplasmic reticulum Ca^2+^ ATPase (SERCA2a) activity was impaired and SERCA2a tyrosine nitration increased compared to wild-type mice [6]. A reduction in SERCA2a activity and expression often leads to poor left ventricular active relaxation and overall left ventricular diastolic dysfunction. In Barth syndrome patients with SERCA2a, expression is often reduced [4] and patients nearly always manifest left ventricular diastolic dysfunction [10]. These findings suggest that SERCA2a abnormalities may be overcome by therapies that target cardiolipin deficiency typically observed in patients with Barth syndrome, particularly those manifesting HFpEF.

In a canine model of HFrEF, SERCA2a activity and protein levels were shown to be significantly decreased in the left ventricular myocardium compared to normal dogs but were normalized after long-term treatment with elamipretide [9]. The improvement in SERCA2a was accompanied by improved indexes of left ventricular diastolic function. Plasma levels of cytokines (tumor necrosis factor-α and interleukin-6) and C-reactive protein are significantly elevated compared to normal baseline levels in dogs with microembolization-induced heart failure [9]. Long-term (3 months) treatment with elamipretide normalized all three of these elevated factors in this heart failure model [9]. Plasma mitochondrial DNA damage-associated molecular patterns (DAMPs), specifically within the COX1 and ND1 genes, are markedly elevated in dogs with heart failure compared to normal. Long-term treatment with elamipretide normalized plasma levels of both COX1 and ND1 DAMPs in this model [4]. These observations of long-term therapy suggest that elamipretide is capable of reversing abnormal cellular and molecular signaling that gradually develops during the development of the heart failure state.

The effects of elamipretide on diastolic left ventricular function have also been examined in a porcine model of renovascular hypertension that manifests HFpEF, as evidenced by preserved LVEF, left ventricular hypertrophy, poor left ventricular relaxation, reduced SERCA2a activity and expression, and reduced phospholamban phosphorylation at serine 16 [8]. Long-term (3 months) therapy with elamipretide normalized mitochondrial respiration; mitochondrial calcium tolerance and permeability pore opening; mitochondrial membrane potential; calcium ATPase activity (SERCA2a); maximum rate of ATP synthesis; mitochondrial complex I and IV activities; reduced cytochrome c release into the cytosolic compartment; and reduced ROS formation. Left ventricular relaxation was improved and cardiomyocyte hypertrophy reduced. These results support the use of elamipretide as a potential therapy for patients with Barth syndrome that manifest a HFpEF phenotype.

#### Durability of action of elamipretide

Therapeutic compounds such as beta-blockers or ACE inhibitors are considered to have “durable effects” in heart failure if the elicited benefits remain evident and dissipate only gradually, within days or weeks, after the withdrawal of therapy. Stated differently, durability reflects sustained pharmacodynamic functional benefits that outlast the pharmacokinetic elimination of the compound. This is in contrast to therapeutic compounds such as the beta_1_-receptor agonist, dobutamine, whereby the elicited benefits dissipate rapidly, often within minutes of withdrawal of therapy. This therapeutic durability on the heart and circulation is often ascribed to a compound the property, during its chronic use, that actively elicits structural, functional, and biochemical benefits which could potentially alter disease trajectory.

**Fig. 2:**
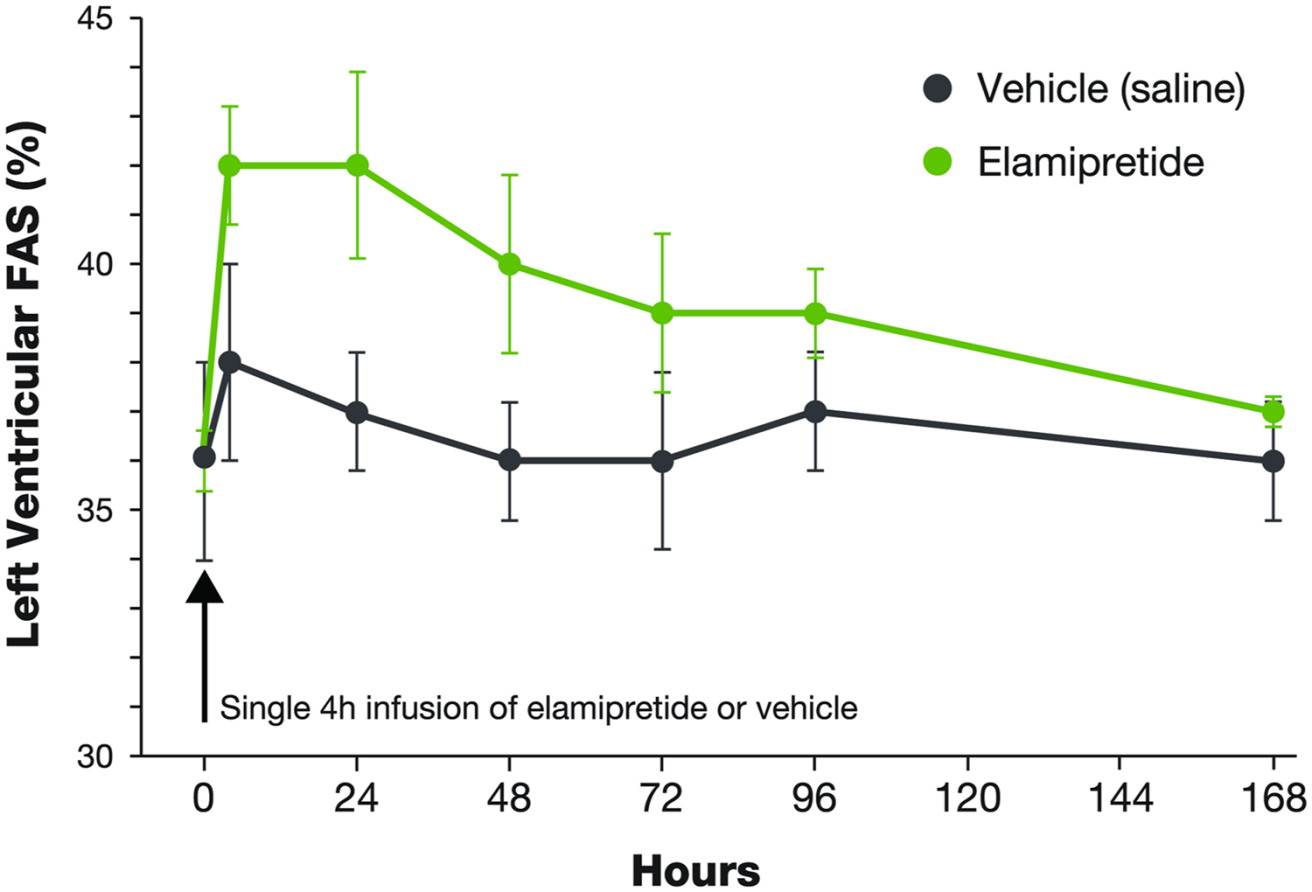
Durability of left ventricular function following a single dose of elamipretide in failing canine hearts [Sabbah personal observation]. In a canine model of chronic heart failure, a single dose of elamipretide resulted in favorable changes in left ventricular fractional area of shortening, the effect of which took 7 days to completely disappear (i.e., beyond the 4-h half-life of elamipretide). Abbreviation: *FAS*, fractional area of shortening

**Fig. 3:**
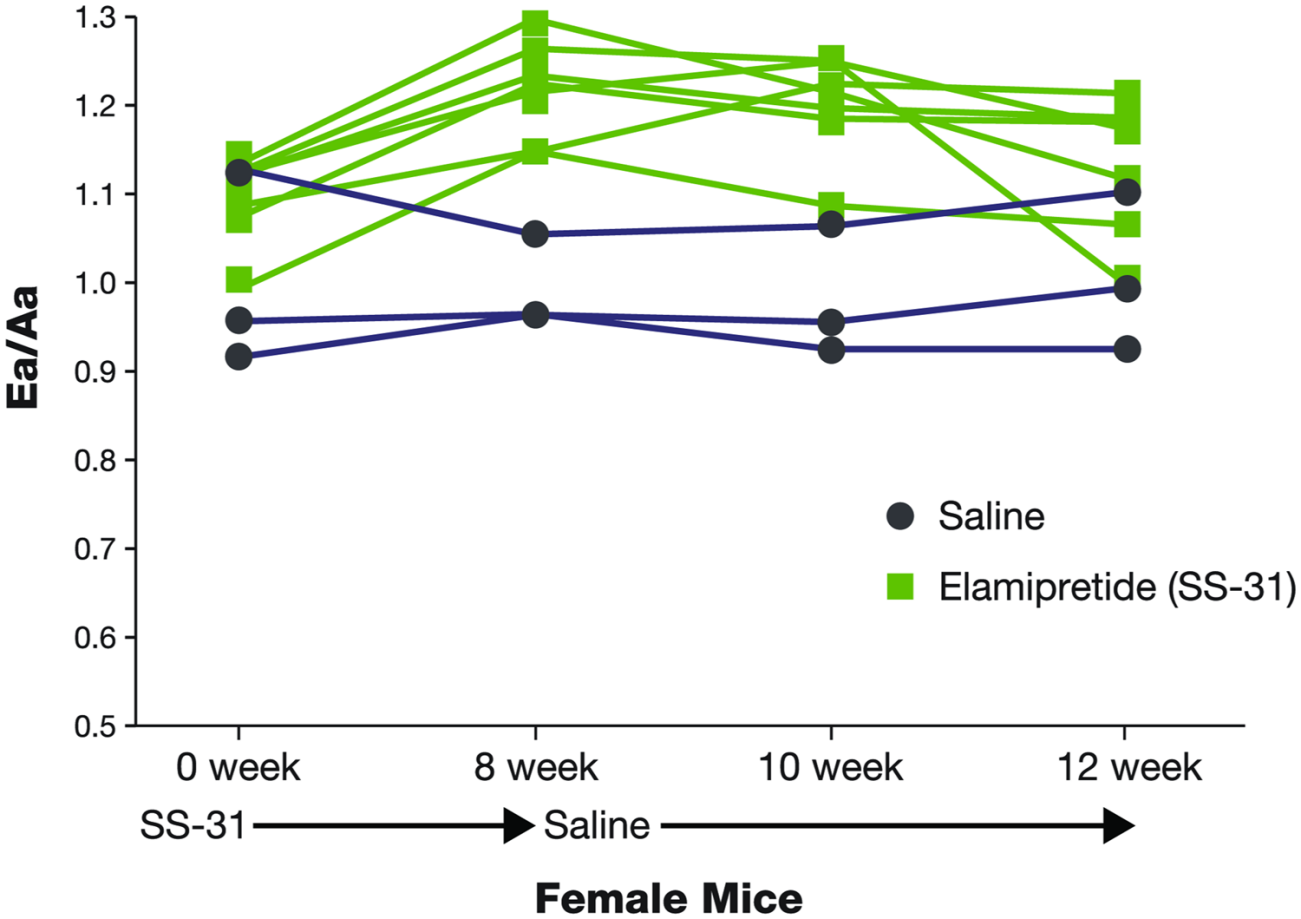
Durability of elamipretide-induced cardiac benefit in mice with diastolic dysfunction [62]. In old mice with LV diastolic dysfunction, treatment with elamipretide for 8 weeks normalized diastolic functional deficit, as evidenced by an increase in Ea/Aa. Improvement in diastolic function was associated with normalization of mitochondrial proton leak, reduced mitochondrial ROS, and reduced protein oxidation overall as well as at the cellular level. Exercise tolerance also improved. Ea/Aa improvement was maintained for up to 2 weeks after withdrawal of elamipretide. Used with permission from [62]. Abbreviations: *Ea/Aa* ratio of early-to-late diastolic mitral annulus velocities, *ROS* reactive oxygen species

In a canine model of chronic heart failure, a single dose of elamipretide resulted in favorable changes in left ventricular fractional shortening that took nearly 7 days to dissipate following dosing, i.e., beyond the 4-h half-life of elamipretide (Fig. 2) [Sabbah, personal observation]. Furthermore, in old (24 months of age) mice with left ventricular diastolic dysfunction as evidenced by a reduced ratio of early-to-late diastolic mitral annulus velocities (Ea/Aa) and an increased (poorer) myocardial performance index, an 8-week treatment with elamipretide normalized the diastolic functional deficit with an increase in Ea/Aa [64]. The improvement of diastolic function was associated with normalization of mitochondrial proton leak, reduced mitochondrial ROS, and reduced protein oxidation overall as well as at the cellular level. In these old mice, exercise tolerance on a treadmill running test was also improved with elamipretide, and a regression of age-related cardiac hypertrophy was observed at necropsy. To evaluate the durability of the elamipretide-induced cardiac benefit, cardiac function was monitored for 4 weeks after the end of treatment. An improvement of Ea/Aa was maintained for up to 2 weeks after withdrawal of elamipretide but dropped by approximately 50% 4 weeks after treatment was stopped (Fig. 3) [64].

Whereas drug withdrawal–type studies, such as those described above, are very helpful in addressing issues related to the “durable effects” of a given cardioactive drug, they are not always possible or appropriate as part of translational preclinical studies or clinical trials. Nonetheless, supportive evidence for establishing the potential of “durable effects” of a cardioactive drug can be ascertained through understanding the mechanism of action of the drug in question. Elamipretide, for instance, selectively targets the mitochondria and directly impacts function of the inner mitochondrial membrane through its association with cardiolipin. This action of elamipretide directly improves myocardial energetics but does not have any direct effects on left ventricular remodeling at the global or cellular levels. Nevertheless, it has been previously shown that long-term monotherapy with elamipretide in a canine model of chronic heart failure, through its action on mitochondria, also leads to improved left ventricular systolic and diastolic function and prevention of progressive left ventricular enlargement [9]. The magnitude of improvement was similar to that observed in the same canine heart failure model after long-term therapy with ACE inhibitors and/or beta-blockers [65]. These benefits occurred alongside normalization of mitochondrial function as evidenced by an improved rate of ATP synthesis and reduced ROS formation. These improvements in histomorphometric measures of left ventricular structural remodeling observed after treatment with elamipretide are similar in magnitude to those seen in this canine heart failure model after monotherapy with ACE inhibitors, angiotensin II receptor blockers, and beta-blockers [65–68] and are not likely to immediately subside if elamipretide therapy is withdrawn.

Elamipretide has also been shown to elicit structural improvement to mitochondria and its constituents that are not likely to immediately dissipate upon withdrawal of elamipretide. The improvement in mitochondrial function following long-term therapy with elamipretide leads to normalization of the mitochondrial inner membrane structure and function, including normalization of cardiolipin and the enzymes responsible for its synthesis and remodeling, and normalization of activities of the various ETC complexes [4, 9, 27]. Normalization of cardiolipin plays an important role in cristae formation, activity of respiratory complexes, organization of the respiratory complexes into supercomplexes for oxidative phosphorylation, mitochondria fusion, and mitochondria DNA stability and segregation [69–72]. Again, these beneficial corrections of mitochondrial structural and functional components are not likely to rapidly dissipate upon withdrawal of elamipretide therapy [71, 73].

Studies in non-cardiac models of disease also support the notion that chronic therapy with elamipretide has “durable organ structural effects.” In rats exposed to 45 min of bilateral renal ischemia and followed for 1 month, glomerular and peritubular capillary rarefaction, macrophage infiltration, fibrosis, mitochondrial degeneration, mitophagy, and deformed foot processes in podocytes were observed. When rats were then treated with elamipretide for 6 weeks (i.e., starting 1 month after ischemia), elamipretide preserved mitochondrial integrity, restored glomerular capillaries and podocyte structure, and attenuated glomerulus sclerosis and interstitial fibrosis [74].

#### Sequence of biological events leading to amelioration of left ventricular remodeling

**Fig. 4:**
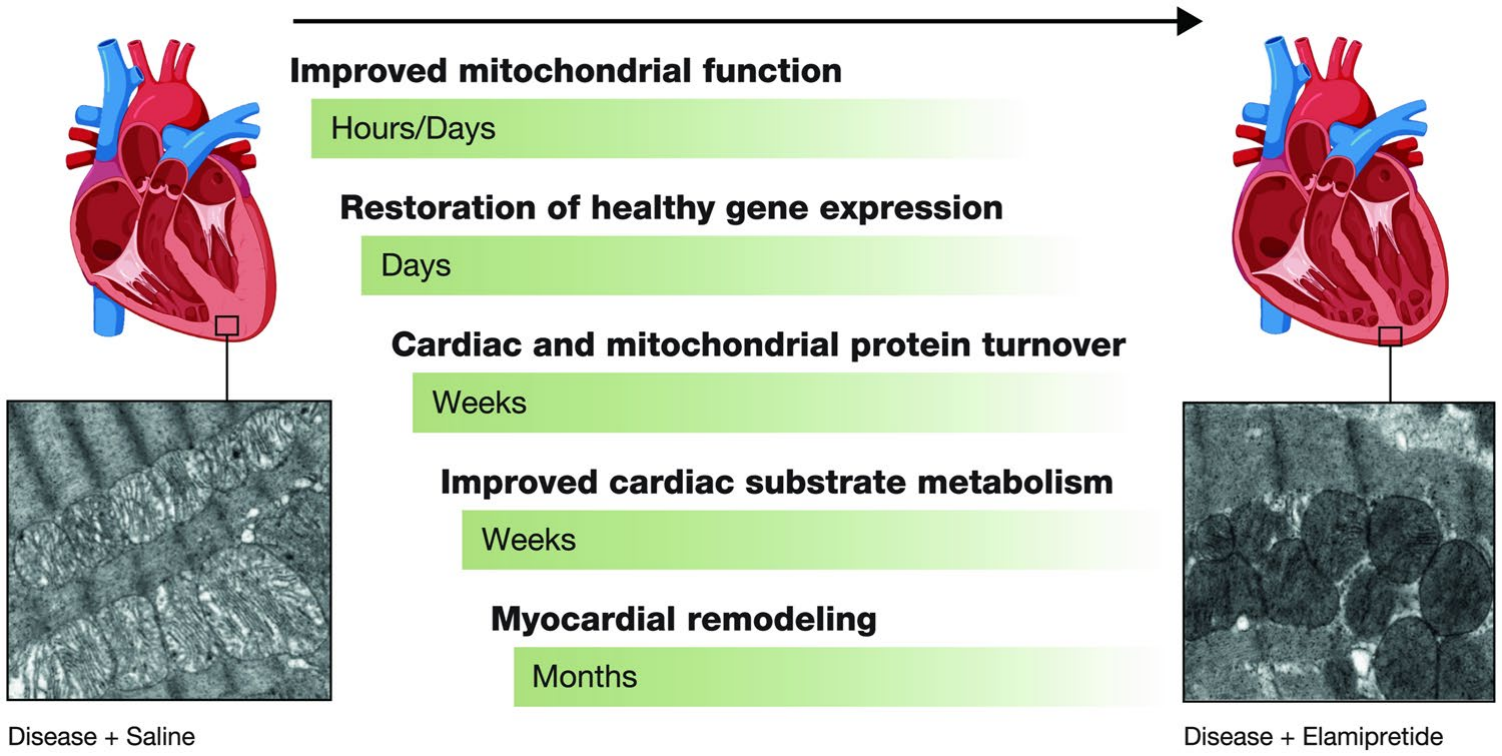
Time course of cardiac changes following treatment with elamipretide [56]. Chronic therapy with elamipretide, which selectively targets abnormal mitochondrial function of the failing heart, can ultimately lead to durable functional and structural improvements of the left ventricle as a whole. Used with permission [56]

There is a good scientific basis for the rationale that chronic therapy with a drug, such as elamipretide, that selectively targets abnormal mitochondrial function of the failing heart can ultimately lead to durable functional and structural improvements of the left ventricle as a whole (Fig. 4) [58]. In the case of the failing or cardiomyopathic heart, improvement of mitochondrial function can occur within hours or days of initiating elamipretide therapy [60, 61]. This rapid improvement in myocardial energetics can, within days or weeks, lead to improved expression of key genes that trigger the synthesis of proteins essential to the recovery of mitochondria and cardiomyocyte toward a more normal phenotype.

In dogs with heart failure, for instance, chronic (3 months) treatment with elamipretide resulted in normalized expression of proteins involved in key pathways such as SERCA2a, peroxisome proliferator-activated receptor co-activator-1α, mitochondrial fission and fusion, cardiolipin synthesis and remodeling, and mitochondria cristae formation among others [9, 27]. Because proper protein turnover in mitochondria and cardiomyocytes requires weeks and possibly months [68], the expectation for restoration of near-normal mitochondria and cardiomyocyte structure and function will occur weeks or even months after initiating therapy. Only then can one expect a drug such as elamipretide to show benefits in preventing or reversing left ventricular remodeling or in improving exercise tolerance in patients with chronic heart failure.

In dogs with chronic heart failure (3 months), therapy with elamipretide resulted in the prevention of progressive left ventricular remodeling evidenced by prevention of progressive left ventricular dilation globally, and by reduced cardiomyocyte hypertrophy and interstitial fibrosis and by increased capillary density at the cellular level [9]. As alluded to earlier, these structural changes of the left ventricular chamber and left ventricular myocardium are similar to those seen in the same dog model of heart failure after chronic therapy with an ACE inhibitor, an angiotensin II receptor blocker, or a beta-blocker, and are part and parcel of the acknowledged durability of these drugs in heart failure.

### Treating Barth syndrome with elamipretide: clinical insights from the TAZPOWER trial

**Fig. 5:**
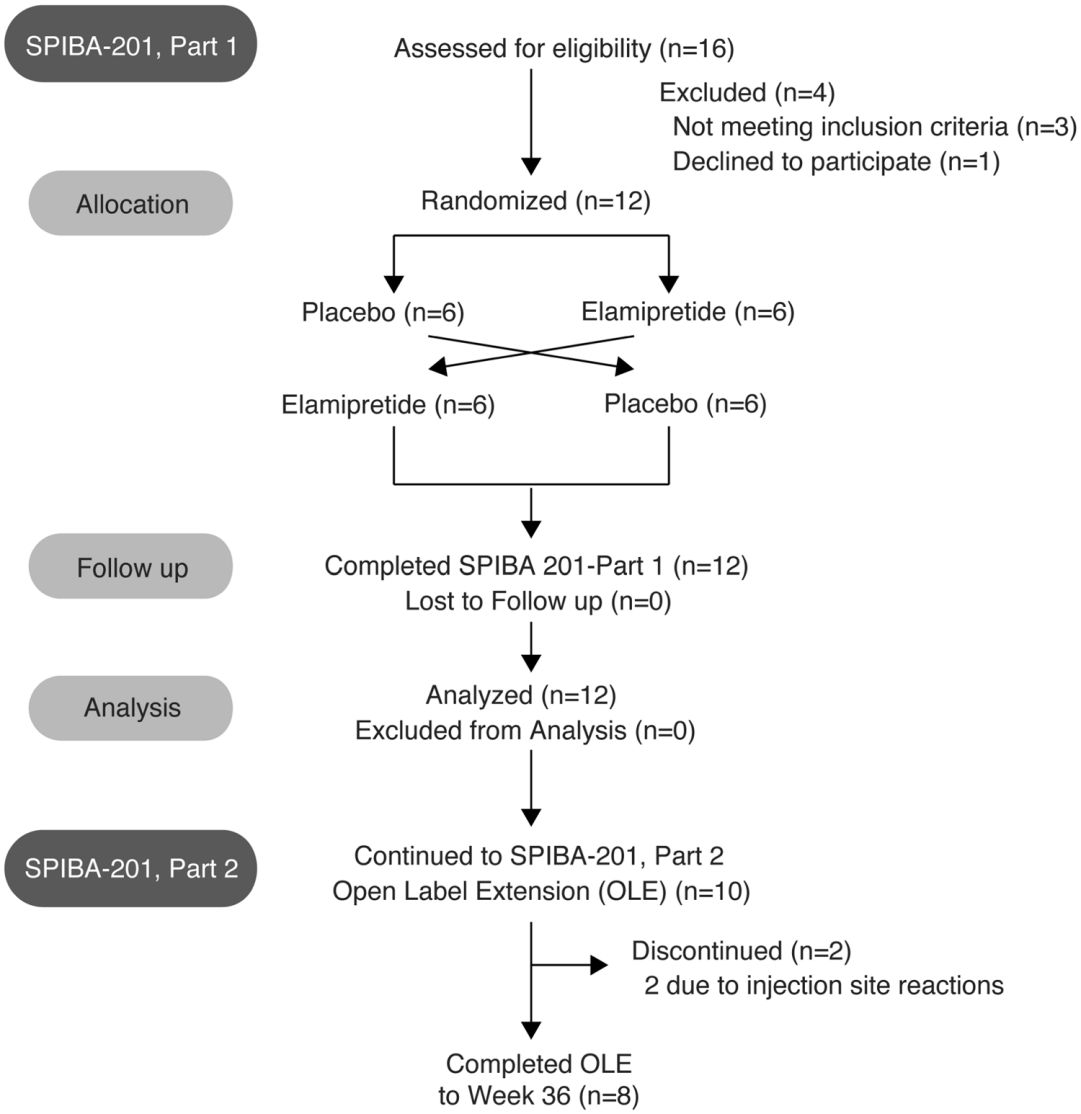
Flow of Barth syndrome patients in the TAZPOWER (SPIBA-201) Trial [16]. Used with permission from [16]

TAZPOWER is a phase 2, randomized, double-blind, placebo-controlled, crossover trial of elamipretide followed by a long-term, open-label treatment extension of elamipretide in patients with genetically confirmed Barth syndrome [16]. The controlled part of the study is complete, while the long-term extension is ongoing. TAZPOWER was designed to evaluate the efficacy and safety of elamipretide in patients with Barth syndrome. Twelve patients (mean age 19.5 years, range 12–35 years) with genetically confirmed Barth syndrome were initially enrolled. The baseline demographic and clinical characteristics of this population are summarized in Table 2. The cardiac phenotype of these patients was hypertrophic cardiomyopathy characterized by lower-than-normal left ventricular end-diastolic and end-systolic volumes, and normal LVEF with reduced stroke volume. In the initial, controlled part of the trial, patients were randomized to once-daily subcutaneous injection of elamipretide 40 mg or placebo for 12 weeks followed by crossover to the alternate treatment after a 4-week washout. In the second part of trial, ten of the 12 patients continued to receive subcutaneous elamipretide 40 mg once daily for up to an additional 168 weeks: eight of the ten patients have completed an additional 36 weeks’ treatment during the open-label extension. A flow chart for patient participation in the trial is shown in Fig. 5.

**Table 2:** Baseline demographic and clinical characteristics of Barth syndrome patients enrolled in the TAZPOWER Trial [16]

Characteristic	Value
Mean age, y (range)	19.5 (12–35)
Male, *n* (%)	12 (100)
Race, *n* (%)	
White	11 ()
Multiracial	1 ()
Ethnicity, *n* (%)	
Not Hispanic or Latino	12 (100)
Hispanic or Latino	0
Mean height, cm (range)	167.3 (150.4–187.7)
Mean weight, kg (range)	50.8 (31.4–85.9)
BMI, kg/m^2^ (range)	17.6 (13.6–24.4)
6MWT, m	395.5
Mean BTHS-SA total fatigue score	8
Mean 3D LVEDV, *Z*-score (SD)	–2 (1.34)
Mean 3D LVSV, *Z*-score (SD)	–1.84 (1.53)
Mean EF, % (SD)	60.6 (4.0)

Used with permission from [Thompson et al. 16]

*6MWT* 6-min walk test, *BTHS-SA* Barth Syndrome Symptom Assessment scale, *BMI* body-mass index, *EF* ejection fraction, *LVEDV* left ventricular end-diastolic volume, *LVSV* left ventricular stroke volume, *SD* standard deviation, *y* year

There were no statistically significant improvements in primary and secondary study endpoints after 12 weeks’ treatment with elamipretide compared to placebo. However, there were statistically significant improvements compared to baseline after completion of 36 weeks’ treatment with elamipretide during the open-label extension (i.e., a total of 48 weeks’ treatment). There was a significant (*p* = 0.020) increase of 95.9 m in 6-min walking time, which was equivalent to a 25% improvement. Additional outcome measures showed improvement at week 36 of the open-label extension including Barth Syndrome Symptom Assessment total fatigue scale score (*p* = 0.030) and muscle strength by hand-held dynamometry (*p* = 0.001). Cardiac function was also determined by echocardiography. Cardiac dysfunction has been shown to be a primary cause of early mortality in patients with Barth syndrome and improvements in LVEDV and left ventricular stroke volume are major determinants of peak exercise capacity in patients with hypertrophic cardiomyopathy. Elamipretide treatment provided a 16% improvement in average left ventricular stroke volume indexed to body surface area from baseline (30.5 ml/m^2^) to the end of the open-label extension (35.3 ml/m^2^). There was a significant (*p* < 0.01) trend of an increase in stroke volume over time using a slope model of individual regression lines for each patient to determine the consistent change in stroke volume index over the course of exposure. Mean stroke volume *Z*-scores increased from − 1.38 at baseline to − 5.6 at week 36 of the open-label extension.

Elamipretide was generally well tolerated with the majority of adverse events being mild to moderate in severity. The majority of adverse events were injection site reactions, including erythema, pain, and pruritus. Two patients discontinued during the open-label extension because of injection site reactions.

## Conclusions

Elamipretide has been shown to improve myocardial energetics through its direct action on the mitochondrial inner membrane through its association with cardiolipin. Multiple studies have shown that the improvement in myocardial energetics as a result of therapy with elamipretide elicits long-term improvements of left ventricular systolic and diastolic function, and, importantly, a prevention or reversal of maladaptive global and cellular left ventricular chamber remodeling. Promising results with elamipretide across various animal models of heart disease are especially encouraging for heart failure and cardiomyopathies in general, including Barth syndrome. Long-term therapy with elamipretide not only normalizes mitochondrial ultrastructure, dynamics (fission and fusion), and function but also appears to have a positive residual lasting footprint on the heart failure/cardiomyopathic phenotype that goes beyond myocardial energetics. Indeed, the benefits extend to improvements of myocardial structure and function that are durable and not likely to immediately dissipate upon withdrawal of therapy. The US Food and Drug Administration has granted Fast Track and Orphan Drug designations for elamipretide in the treatment of patients with Barth syndrome. The clinical results seen in patients with Barth syndrome treated with elamipretide support the potential use of this drug for the clinical management of this rare disease syndrome [16].

## Data Availability

Not applicable.
